# Socioeconomic and substance use changes in emerging adults and their relationship with mood disorders in a population-based cohort

**DOI:** 10.3389/fpsyt.2022.932484

**Published:** 2022-08-24

**Authors:** Clarisse de Azambuja Farias, Taiane de Azevedo Cardoso, Marielle Moro da Silva, Francesca D’Angelo, Thaise Campos Mondin, Luciano Dias de Mattos Souza, Ricardo Azevedo da Silva, Flavio Kapczinski, Karen Jansen, Pedro V. S. Magalhães

**Affiliations:** ^1^Graduate Program in Psychiatry and Behavioral Sciences, Hospital de Clínicas de Porto Alegre, Universidade Federal do Rio Grande do Sul, Porto Alegre, Brazil; ^2^Graduate Program in Health and Behavior, Universidade Católica de Pelotas, Pelotas, Brazil; ^3^Department of Psychiatry and Behavioral Neurosciences, McMaster University, Hamilton, ON, Canada; ^4^Faculty of Medicine, Hospital de Clínicas de Porto Alegre, Universidade Federal do Rio Grande do Sul, Porto Alegre, Brazil

**Keywords:** emerging adults, mood disorder, substance use disorder, social roles, economic status, longitudinal studies

## Abstract

In this report, we aim to assess the interaction of bipolar disorder and major depressive disorder with the evolution of social roles, economic classification, and substance misuse in emerging adults. This is a longitudinal population-based study (*n* = 231 at baseline), in which participants were reassessed at a mean of 5 years after baseline. A structured clinical interview was used to diagnose the participants with bipolar disorder and major depression; a control group without mood disorders was included. Men with mood disorders were less likely to be married in the beginning of the study and less likely to work in the follow-up. Women with major depression were less likely to study and more likely to be in a lower economic class at the beginning of the study. In comparison, women with bipolar disorder were less likely to live with their parents and more likely to live with their children in the first wave of the study. Substance misuse was more likely in people with mood disorders, especially in men, and women with bipolar disorder had the highest likelihood in the follow-up. Albeit longitudinal analyses were limited by a possibly insufficient sample size and mediating mechanisms for change, such as stigma, were not explored, the study suggests sex-related specificities regarding the change in social roles and substance use in people with mood disorders. Emerging adults, especially those with mood disorders, are in a period of change and instability and at a greater risk for substance use and abuse.

## Introduction

The way people live the period of their lives between 18 and 29 years old has changed substantially in the last 50 years. Demographic trends of a longer period of education and an older age to get married and have children have led to the proposal for a new developmental period, emerging adulthood (18–29 years old) (1, 2). This proposal arises as an attempt to describe what it means to be an adult and what defines the transition to adulthood, with cultural influences that change over time. This period is not just a brief transition, but a period of many changes and instabilities in different areas of life, a long phase before achieving stable adulthood (2). As a result, specific characteristics, even if not exclusive to the period, have been proposed for the emerging adult life, such as identity explorations, instability (romantic relationships, work, residence), focus on oneself (less daily social roles and obligations to others), “feeling in-between” (neither teenager nor adult) and possibilities or optimism (about the future; work and economic conditions) (1, 2). While the important experiences that make up this phase can be exciting, they can also be confusing and challenging. Involuntary changes, reduced social support, and high self-demand can all be associated with depressive and anxious symptoms in emerging adults (2).

Thus, some more prominent developmental challenges for the emerging adult age, such as the transition process from living with parents to no longer living with them, starting the journey to higher education, following a professional path, seeking financial autonomy, and finding a life partner, may be associated with the incidence of mental health problems at this stage (2). There are also aspects of brain development that make emerging adults especially vulnerable to mental disorders, such as the excessively rapid synaptic pruning (3), possibly having an interaction with the environment (4). As a result, the very fast synaptic reduction can increase the sensitivity to stress and turn the individual vulnerable to mental disorders (4). Multiple mental disorders are more prevalent during the emerging adult stage, especially mood disorders (5, 6). The onset of mental illness during emerging adulthood has the potential of adversely impacting development and identity formation (7).

Considering the impairments associated with major depressive disorder and bipolar disorder, such as functional impairment (family, social and occupational areas) (8–13), cognitive impairment (12, 14–17), poor quality of life (10), and the changes and uncertainties of the emerging adult phase, it is of extreme importance to monitor emerging adults with mental disorders. It is in this stage of life that there is the highest probability for the onset of bipolar disorder (18). This period can impose multiple challenges, from symptom management to maintaining functioning and relationships (19). In addition, recent evidence suggests that most subjects diagnosed with bipolar disorder transition to multiple episodes within 5 years of the onset of the disorder (20, 21).

Another point of convergence between the onset of certain mental disorders and this stage of life is substance use and misuse. Emerging adults are more likely to present substance abuse or dependence when compared to young adults (22). People with mood disorders have a high prevalence of comorbidity with substance use disorder (23–27). Post and Kalivas (28), postulated that cross-sensitization between stressors, episodes, and inadequate use of substances contribute to the progression of bipolar disorder (28).

The few longitudinal studies that have investigated the evolution of emerging adults with mental disorders tend to be based on clinical samples or of limited population generalizability (29, 30). In addition, there is a trend in the mood disorder literature to investigate only clinical outcomes, such as symptoms, mood episodes, and relapses. The investigation of the broader biopsychosocial development, such as leaving the parent’s house, the transition from study to work, and the constitution of new families has been less often investigated. Thus, the objective of the present study was to assess the interaction of bipolar disorder and major depressive disorder with social roles, economic classification, and substance misuse in emerging adults, in a longitudinal population-based study.

## Materials and methods

### Design and participants

In this cohort, we followed up matched cases and controls nested in a population-based sample. Full descriptions of the original study have been published elsewhere (31–33). Briefly, initially, 1,560 participants aged between 18 and 24 were assessed in the period from 2007 to 2009; sample selection was performed by clusters. Eighty-nine census-based urban sectors were randomly selected from the city of Pelotas, in South Brazil (34). For this nested sample, every participant with a past or current history of a manic or hypomanic episode from the population-based study was included at baseline. Ninety-three individuals met this criterion by Mini International Neuropsychiatric Interview (MINI) (35, 36). Two comparison groups were recruited. People without any history of mood episodes were randomly selected and frequency-matched for sex, age, and economic situation, i.e., a healthy control sample. We also recruited a group with a current depression but no past history of (hypo) mania. Other mental disorders or clinical morbidity, including substance use, were not a reason for exclusion. We were able to obtain data on 231 subjects (83% of the intended sample) at baseline. The whole matched sample of cases and controls further underwent the Structured Clinical Interview for DSM-IV (SCID) (37, 38) to confirm diagnoses and improve reliability, and this is the group-defining criterion for this study. After SCID diagnoses, the final baseline sample consisted of 93 participants without any history of mood episodes, 83 participants with major depression, and 55 with bipolar disorder (33 type I and 22 type II) (31, 33).

Second wave data were collected between 2012 and 2014. The 231 participants were invited to return for a follow-up assessment after a mean of 5 years post-baseline (39, 40). Master’s or Ph.D. level psychologists again evaluated current mood status using the Mini International Neuropsychiatric Interview (MINI-PLUS) (35, 36), and in cases of diagnostic doubt, reassessments were conducted with the SCID (37, 38). Those who consented to participate in the study signed an informed consent form. Participants who had a psychiatric disorder at baseline, as well as at the 5-year follow-up, were referred for treatment at the Clinic of Research and Extension in Mental Health of the *Universidade Católica de Pelotas*. The Research Ethics Committees of the *Universidade Católica de Pelotas* and *Hospital de Clínicas de Porto Alegre* approved the study.

### Instruments

Participants answered a questionnaire with sociodemographic variables, including age, sex, having a partner, living with offspring, and occupational status (work and study). Economic status was defined according to the criteria of the Brazilian Association of Research Companies (41). This instrument measures economic classification through the accumulation of material goods (color television, radio, bathroom, automobiles, monthly employee, washing machine, VCR/DVD, refrigerator, freezer—independent appliance or part of the duplex refrigerator) and the level of schooling of the householder. The sum of the points results in a total score, classified into five groups (A, B, C, D, and E), where “A” refers to the highest economic classification and “E” to the lowest, consistent with previous research, we dichotomize participants into two categories, A/B/C (high) and D/E (low). Substance use disorders (tobacco, alcohol, and other substances) were assessed with the Alcohol, Smoking and Substance Involvement Screening Test (ASSIST) (42, 43), with a cutoff of four. The ASSIST was used at baseline and follow-up assessment (39, 44, 45).

### Statistical analysis

We use chi-squared statistics to investigate differences in proportions. A generalized estimating equations (GEE) model with Poisson distribution and robust standard errors was used to investigate differences in proportions between diagnostic groups and from baseline to endpoint. The model includes diagnosis, sex, follow-up wave, and interactions between group and sex and group and wave.

## Results

Two hundred and seven (89.6%) out of the 231 individuals assessed at baseline were re-assessed at follow-up. The baseline sample consisted of 158 (68.4%) women. More than half of the sample identified themselves as having white skin color (153; 66.2%). The average age at baseline was 22.04 (s.d. 2.18), and the average time between the two assessments was 5 years (Table 1).

**TABLE 1:** Sample characteristics according to baseline diagnosis.

Variables	Bipolar disorder (*n* = 55)	Major depression (*n* = 83)	Control (*n* = 93)
Sex^a^*			
Male	14 (25.5)	19 (22.9)	40 (43.0)
Female	41 (74.5)	64 (77.1)	53 (57.0)
**Skin color^a^**			
White	36 (65.5)	52 (62.7)	65 (69.9)
Non-white	19 (34.5)	31 (37.3)	28 (30.1)
**Age (years)^b^**	21.82 ± 2.26	21.78 ± 2.00	22.40 ± 2.26
**Economic classification (ABEP)*^a^***			
Upper (A + B)	14 (25.5)	24 (28.9)	35 (37.6)
Middle (C)	33 (60.0)	40 (48.2)	46 (49.5)
Lower (D + E)	8 (14.5)	19 (22.9)	12 (12.9)
**Years of education^b,c^**	8.82 ± 3.56	8.91 ± 2.75	9.69 ± 3.18
**Medical illness^a,d^**			
No	29 (52.7)	55 (67.1)	67 (72.0)
Yes	26 (47.3)	27 (32.9)	26 (28.0)
**Substance abuse/dependence (ASSIST)^a^**			
Tobacco**	25 (45.5)	32 (38.6)	22 (23.7)
Alcohol	21 (38.2)	32 (38.6)	24 (25.8)
Any illicit drug	9 (16.4)	19 (22.9)	9 (9.7)

ABEP, *Associação Brasileira de Empresas de Pesquisa*; ASSIST, Alcohol, Smoking and Substance Involvement Screening Test.

^a^Absolute and relative frequencies (%), Chi-square test.

^b^Mean and standard deviation, ANOVA test.

^c^Missing (n = 31).

^d^Missing (n = 1).

*p = 0.009; **p = 0.015.

Considering the entire sample in the follow-up assessment, there were significantly more people working (OR = 4.46; 95%CI: 2.97–6.70; *p* < 0.001), living with a partner (OR = 2.29; 95%CI: 1.54–3.40; *p* < 0.001), who were living with their children (OR = 2.51; 95%CI: 1.68–3.74; *p* < 0.001), and fewer people studying (OR 0.41;95%CI: 0.27–0.63; *p* < 0.001) or living with their parents (OR = 0.26; 95%CI: 0.17–0.38; *p* < 0.001). In addition, there were fewer people in the economic classes D or E (lowest classes) (OR = 0.30; 95%CI: 0.15–0.60; *p* < 0.001).

**FIGURE 1:**
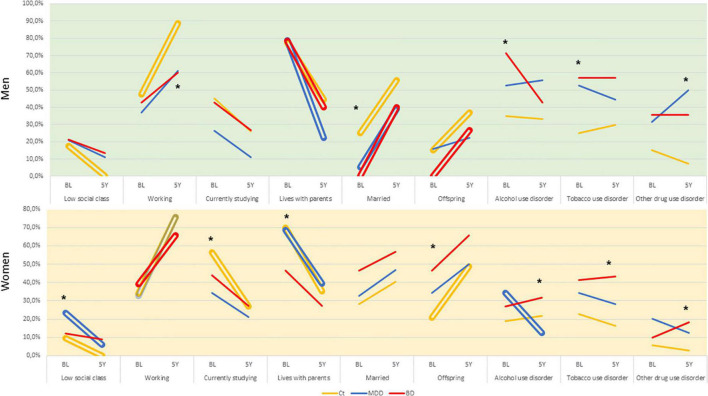
Changes in socioeconomic variables and substance abuse/dependence according sex at baseline and 5-year follow-up in emerging adults with and without mood disorders. Double bars indicate significant within-group changes (*p* < 0.05) and asterisks indicate significant between-group changes (*p* < 0.05).

Several differences were observed between baseline and the 5-year follow-up, as well as between the diagnostic groups. There were changes in working and living, and the explanatory analysis suggested different patterns of change associated with group and sex (Figure 1). Men with mood disorders were less likely to be working at follow-up and to be married at baseline; for substance use disorders, men already had differences in mood disorder groups at baseline. Women with mood disorders had a lower chance of being currently studying at baseline and a higher chance of already having children of their own and not living with their parents at baseline; they were also more likely to present substance use disorders in the 5-year follow-up. All the people in the control group left the lower economic classes, which did not happen in the mood disorders groups.

The GEE models, however, tended to retain only the main effects of time and diagnostic group, with a few interesting exceptions. Having a partner was significantly more likely for women in both mood disorder groups and living with their children was more likely for women with bipolar disorder (Table 2).

**TABLE 2:** Effects of diagnosis, sex, and study wave on socioeconomic variables and substance abuse/dependence in the final model (*n* = 231).

	Low economic class		Lives with parents		Lives with partner		Lives with children		Studying		Working		Alcohol abuse/ Dependence		Tobacco abuse/ Dependence		Any illicit drug abuse/ Dependence	
									
	RR	95%CI	RR	95%CI	RR	95%CI	RR	95%CI	RR	95%CI	RR	95%CI	RR	95%CI	RR	95%CI	RR	95%CI
Major depression	**2.31**	**1.23–4.36**	0.86	0.62–1.18	0.56	0.30–1.07	**2.01**	**1.19–3.40**	**1.92**	**1.04–3.56**	**2.81**	**1.22–6.50**	1.01	0.43–2.40	0.51	0.23–1.14	0.69	0.41–1.17
Major depression*wave	−	−	0.94	0.62–1.42	0.97	0.56–1.67	0.56	0.29–1.07	0.89	0.54–1.47	1.87	0.52–6.81	0.61	0.35–1.06	1.13	0.59–2.19	1.05	0.69–1.60
Major depression*sex	−	−	1.22	0.81–1.85	**2.06**	**1.00–4.24**	0.75	0.36–1.56	0.86	0.37–2.04	1.08	0.29–4.01	1.66	0.69–4.00	1.24	0.52–2.93	1.40	0.88–2.22
Bipolar disorder	1.91	0.91–4.00	0.96	0.69–1.34	0.57	0.27–1.19	1.76	0.99–3.15	**2.04**	**1.05–3.94**	1.99	0.69–5.75	0.68	0.25–1.83	0.92	0.47–1.82	0.87	0.51–1.49
Bipolar disorder*wave	−	−	1.05	0.64–1.71	0.87	0.52–1.46	0.88	0.46–1.71	1.11	0.70–1.76	2.85	0.77–10.47	0.68	0.40–1.15	1.21	0.66–2.23	0.79	0.51–1.21
Bipolar disorder*sex	−	−	0.72	0.43–1.18	**2.81**	**1.22–6.50**	0.89	0.42–1.88	0.99	0.41–2.42	1.05	0.24–4.56	**3.06**	**1.07–8.76**	0.86	0.39–1.87	1.29	0.78–2.14
Study wave	**0.33**	**0.19–0.57**	**0.53**	**0.40–0.71**	**1.75**	**1.21–2.52**	01.03	0.65–1.65	0.93	0.65–1.33	0.49	0.15–1.57	**2.39**	**1.54–3.71**	**0.51**	**0.34–0.77**	**2.05**	**1.57–2.66**
Sex	0.71	0.41–1.22	0.87	0.66–1.15	0.89	0.56–1.42	0.58	0.33–1.04	0.74	0.36–1.55	0.37	0.11–1.23	1.34	0.76–2.37	1.20	0.79–1.82	0.79	0.61–1.03

Bold values represent p ≤ 0.05.

RR, risk ratio; CI, confidence intervals.

## Discussion

This study explored psychosocial changes over an average period of 5 years in a population-based sample of emerging adults. In general, the changes tended to follow an expected pattern—young people left home, many stopped studying and started working, got married, and started a new family. In groups diagnosed with a mood disorder, exploratory analyses suggested some potentially relevant deviations. Many differences were already present at baseline, especially in men regarding substance misuse. Women, especially with bipolar disorder, at baseline were already more likely to have left their parents’ house, to be married, and be living with their own children. For both sexes, there was a significant change in economic class, and none of the controls were in the lower classes at follow-up, which was not true for the groups with mood disorders.

According to Arnett et al. (2), emerging adults, unlike teenagers, have reached physical and sexual maturity, are legally responsible for their actions, and present diversities in their combinations of educational and occupational trajectories. In contrast with young adults in their thirties, most emerging adults have not yet established a stable structure in adult life, with long-term commitments in love and work relationships (2). Even when considering this instability and variability common to the emerging adulthood phase, the changes found between the groups studied here suggest the possibility of significantly different courses for emerging adults with mood disorders. Some findings from the exploratory analyses suggest that women with mood disorders already have more characteristics of young adults at this stage, married and living with children. Men with mood disorders, on the other hand, seemed to be much less likely to be working at follow-up, which possibly already reflects some degree of impairment associated with the disorder.

In this period of so many new challenges, family relationships can have a significant impact on this important journey of emerging adults. In a study conducted with 1,502 undergraduate students, it was found that family plays a fundamental role in the psychological wellbeing of emerging adults. Parental support for autonomy, low levels of behavioral and psychological control were found to be important factors for having a higher level of psychological adjustment among emerging adults (46). In contrast, there is also the impact of a dysfunctional family dynamic, as well as a family history of psychiatric illness. A cross-sectional population-based study found that a family history of mood disorders was associated with mood disorders in emerging adults and childhood traumas. In that study, it was also found that childhood trauma was a mediating factor for the association between a family history of mood disorder and mood disorder in emerging adulthood (47). The phenomenon characterized here may be related to our finding and the history of mental illness in the family is an additional complicating factor in the transition, causing, for example, emerging adults with bipolar disorder to leave their parents’ home earlier to avoid abusive relationships.

In the present study, we found that women at baseline, especially those with bipolar disorder, were already more likely to have left their parents’ home, to be married, and living with children. The literature shows that among subjects with bipolar disorder, women are more likely to marry than men. Lieberman et al. (48) found in their study that among men with bipolar disorder, those who were never married were more likely to have bipolar disorder I and earlier onset of illness compared to married men. Furthermore, it was observed that among women with bipolar disorder, those who were married showed an improvement in the course of the disease, with a reduction in depressive episodes over a 2-year period and a lower cumulative depression severity when compared to those who were not married. However, marriage was not associated with a difference in the course of the disorder among married men, suggesting that women may be more sensitive to the positive effects of social support available in a stable marital relationship (48). In addition, men with mood disorders may present a lower chance of being married, as they are at the beginning of the disorder and face symptoms differently compared to women.

The problematic use of substances may be one of the responsible factors for these trajectories. Emerging adults are in a period of many changes and instabilities, and at a greater risk for substance use and abuse (49, 50). In addition to emerging adults (18–25 years) presenting a higher prevalence of mental health problems and substance use disorders, they also present lower treatment rates (51). In the present study, the prevalence of substance use disorders was greater in participants with mood disorders. We also found that, in addition to the higher prevalence of substance use disorders among men with mood disorders, an increase was observed among women with bipolar disorder at follow-up. According to the literature, there is a higher prevalence of substance use disorders in men in this period, compared to women (22, 51). Men with major depressive episodes are more likely to report alcohol abuse/dependence (22).

Overall, women start using drugs at lower doses than men, but drug use evolves more quickly into addiction, and they face a higher risk of negative health consequences and relapse after abstinence (52). This could be one of the explanations as to why the prevalence of substance use disorders in women with bipolar disorder increased at follow-up. Another possible explanation is that substance use may be less socially acceptable for women than for men. In this case, women who develop a substance use disorder may represent the more severely affected people with a higher risk of psychiatric comorbidity, as mentioned in another study (53). The fact that substance misuse is already installed at this stage implies additional difficulties for the development of emerging adults. These relationships are complex, and substance use can also be related to the onset of mood disorders or to a complicated course of illness (54). It can also be an additional burden that makes it difficult to adapt to new stages of development.

It is in the emerging adult phase that many people first enter into the labor market and face the challenges of being admitted to desired jobs (1), and paid work can be a marker of social inclusion and status (55). In addition, there is a relationship between mental disorders and unemployment, underemployment, temporary leave, and retirement (30, 56–58). Butterworth et al. (59), observed in a longitudinal study that impairments in the mental health state of individuals assessed at baseline, for example, symptoms of depression, were predictors of underemployment in subsequent years. In the present study, we found that men with mood disorders seemed to be less likely to be working in the follow-up. According to the systematic review of Marwaha et al. (55), people with bipolar disorder early in the course of the illness tend to have higher employment rates than later in the course of illness and that employment patterns may differ depending on the stage of the disorder. For people with mental illness, work is perceived as an important component for their recovery (60). As found by Luo et al. (61), the relationship between work and the course of the illness possibly has effects to a greater degree for men than for women with major depressive disorder (61). We can also think that the duration of unemployment is possibly greater for men with mood disorders than for women (59).

Despite possibly being an unstable journey (2), individuals in the emerging adult age tend to initiate a movement toward financial autonomy. However, people with mental disorders often have impairments in their functioning, including in the workplace (8–10, 13, 62–64). This will possibly have an impact on their economic conditions. Hakulinen et al. (57) found in a cohort study (measured years 1988–2015) that earnings for individuals with severe mental disorders were considerably low and that the mean total annual income remained stable between 25 and 52 years for most groups of mental disorders (57). Recently, Cao et al. (65) found a unidirectional effect from depression to financial stress on emerging adults. In addition, a case-control study carried out from records from all individuals (*N* = 50,551) who were hospitalized for severe mental disorders, including bipolar disorder, between 1988 and 2015 in Finland found that, on average, during the year when the first inpatient diagnosis was given, individuals with bipolar disorder were earning 76% of the total income earned by the control group. Five years later they were earning 67% and 10 years later they were earning 66% (30). Similar findings were observed in a longitudinal study conducted in Taiwan, showing that the probability of switching to a person without income is significantly higher in patients with bipolar disorder than in controls over time (29).

In the present study, differences in economic class change may have occurred due to a series of factors related to the mental illness. Mood disorders are associated with impairment of occupational functioning, possibly associated with cognitive difficulties directly resulting from the illness or its treatment (12, 14, 16, 17, 66–68). Furthermore, depression has been associated with an increased risk of unemployment, particularly for those with less education (69). Educational level is an additional predictor of future employment in mood disorders (70). The early onset of mood disorders can have a negative impact on the educational process, often leading to interruption of studies in youth (55). Mental illness can also impair social skills directly relevant to functioning. Close to 30–60% of people with bipolar disorder fail to regain full functioning in the occupational and social domains (71). Another area that can cause harm is the stigma associated with mental disorders (72). Substance use can also play a role in limiting youth performance and opportunities. Due to the low rate of functional recovery in individuals with severe mental disorders, they have a great likelihood of receiving a permanent or temporary disability pension after they are diagnosed. In Finland, after the onset of a severe mental disorder, the disability pension is the main source of income for most individuals with severe mental disorder and their long-term economic wellbeing depends largely on this pension (30). In Brazil, a multicenter study carried out with 2,475 patients with mental disorders, found that 35.6% of the sample received disability benefits or retirement, and 46.6% of the patients had no individual source of income or obtained income that was not related to work activity, such as assistance from the State, pension, assistance from family members (56, 62). Few studies have assessed economic status prospectively.

Arnett’s theory of emerging adulthood has been based mainly on subjects living in developed countries (1). In Brazil, economic and cultural issues can be quite different when compared to developed countries, and this may influence the characteristics of this stage of life, which may be a conceptual limitation to link our findings with a context of this stage. Here, we could not assess the self-stigma and social stigma of the disease, a possible mechanism underlying some of the findings. It is also possible that the study was underpowered to detect some relevant differences between time and sex interactions. Here we also only assessed two time points within this sensitive period. Future cohort assessments should be able to clarify some effects found. Longitudinal studies that monitor the periods of change during this phase may also contribute to the improvement of preventive and treatment actions, especially when it comes to substance misuse. The effect of early treatment was also not assessed here. Future studies with a qualitative approach may also contribute to a greater understanding of the facilitators and barriers present in the development of this phase. Despite these limitations, a major difference of the present study is that it included a community sample of emerging adults and analyzed several social aspects in a longitudinal design.

The trajectories of emerging adulthood in young people with mood disorders that we examined here may suggest pathways potentially associated with impairment, with relevant consequences. In this sensitive period, the acquisition of certain skills is expected, and significant deviations here can lead to impairments in functioning associated with difficulties in the ability to work and develop interpersonal relationships. A marker of emerging adulthood, substance use, can have significant interactions with mood disorders and should be a focus of investigation associated with these impairments. These findings also reinforce the probable impacts that early diagnosis and interventions may have on social, occupational, and education skills and also on the risks of substance misuse during this period.

## Data availability statement

The raw data supporting the conclusions of this article will be made available by the authors, without undue reservation.

## Ethics statement

The studies involving human participants were reviewed and approved by Comitê de Ética e Pesquisa do Hospital de Clínicas de Porto Alegre. The patients/participants provided their written informed consent to participate in this study.

## Author contributions

All authors made substantial contributions to the conception or design of the work, the acquisition, analysis, and interpretation of data for the work, and drafting of the work, revised it critically for important intellectual content, approved the version to be published, and agreed to be accountable for all aspects of the work in ensuring that questions related to the accuracy or integrity of any part of the work are appropriately investigated and resolved.
